# Applying Principles of Evolutionary Biology to Plastic Surgery at an Organizational Level Predicts an Extinction Event

**DOI:** 10.1093/asjof/ojad057

**Published:** 2023-06-26

**Authors:** Ross I S Zbar, Lisa D Taylor, John W Canady

## Abstract

As an organized profession, plastic surgery struggles delivering a clear message regarding scope of practice to patients given the diversity of procedures performed. Whereas granting licensure to practice medicine resides with governmental bodies, certification rests with organizations. However, certification is not required to practice plastic surgery. Since plastic surgery operationalizes techniques rather than working within a defined body organ, competition for patients is intense. Mapping territorial interactions between healthcare providers while parsing taxonomy elucidates individual, community, organizational, and governmental levels, creating various selection pressures. Applying evolutionary biology as a framework predicts the termination of plastic surgery over time as a unique specialty. An entirely new domain, Restorative Healthcare, is proposed which circumvents an extinction outcome.

On an organizational level, plastic surgery struggles with—and is losing—the battle for branding. What exactly is plastic surgery and who can perform it? Is it a cosmetic or reconstructive endeavor or perhaps both? Going to the homepage of the American Society of Plastic Surgery (ASPS) website, visitors are faced with two large Uniform Resource Locator (URL) link buttons labeled “Cosmetic Surgery Selector” and “Reconstructive Surgery Selector.”^1^ This mixed messaging has been present since the early days of the specialty in the 19th century. The difference between Cosmetic or Reconstructive endeavors is critical not only for branding but also for reimbursement with the growth of commercial health insurance. To update its messaging, a name change was launched in 1999 by the American Society of Plastic and Reconstructive Surgery, where the “Reconstructive” moniker was dropped “to convince people that plastic surgeons and reconstructive surgeons are one and the same.”^2^ Twenty years later, this struggle continues.

To better understand the interactions between various professional organizations, thematic relationships can be graphically mapped.^3,4^ Applying this social science exercise with an evolutionary bias^5‐7^ creates a novel methodology, which we label the sociobioecological perspective. Applying this technique illustrates that current trends will lead to the extinction of plastic surgery as a distinct species on an organizational level due to severe competition.

## WHO CAN PERFORM PLASTIC SURGERY AND WHY?

**Figure 1.**
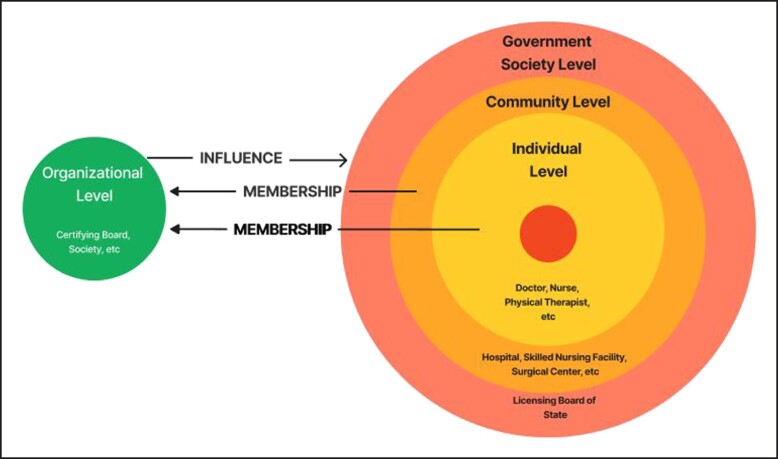
Illustration of the sociobioecological levels and their interrelationships in the practice of plastic surgery.

The structure of healthcare licensure in the United States varies between states based solely on the powers established by federal and state constitutions with a factor of interpretation from the courts. Individual healthcare providers are granted licensure to practice their craft based on processes policed by the Executive Branch but defined within the Legislative Branch of each state government. Healthcare fields, such as medicine (commonly called “medicine and surgery”), dentistry, nursing, optometry, podiatry, physical therapy, or barber/aesthetician are legally defined at the government society level. For example, legislated qualifications for those licensed as physicians who practice medicine mandate earning an MD or DO degree. But professional licensure also applies to other fields of healthcare as defined by the government. Within the last few decades, multiple healthcare-related professions have earned recognition and consequently require licensure, such as dietetics, social work, and genetic counseling. Reasons for this growth include both consumer protection as well as reimbursement from Medicare, Medicaid, and other third-party payers. Additionally, new fields related to well-being emerged that similarly require licensure in many states, these include massage therapy and athletic training. Meanwhile, scopes of practice are expanding and overlapping. For example, those licensed by the Montana Board of Barbers and Cosmetologists may perform microdermabrasion but not injectables^8^; whereas in Pennsylvania microblading is outside the scope of the cosmetology field.^9^ On the community level of this delivery model, a hospital (which is also licensed by the state) grants privileges to individual members of the staff to practice their craft based on guidelines and processes established within the facility's by-laws but which must comply with state laws. Meanwhile, these by-laws are influenced by accreditation requirements established as conditions of reimbursement from payers as well as organizational messaging.^10^ These complex relationships are mapped in Figure 1 using sociobioecological methodology.^11^

On the government society level, the executive bodies of each state granting permission to practice in a field are known colloquially as licensing boards (eg, Board of Medicine) but the actual name of these bodies varies from state to state. Examples include the Georgia Composite Medical Board, Iowa Board of Medicine, New York State Department of Education overseeing the Office of Professions, or the New Jersey Division of Consumer Affairs overseeing the State Board of Medical Examiners. The licensing boards on the state level must not be confused with those boards granting certification in a particular field of medicine (eg, American Board of Plastic Surgery, ABPS) on the national level. There are numerous other certifying boards in other fields as well. For example, the American Board of Podiatric Medicine, the American Board of Optometry, or the National Commission on Certification of Physician Assistants all certify and require maintenance of certification from its members. Nevertheless, it is the granting of the license at the government society level by the state that formally dictates the scope of practice for a healthcare practitioner—not certification by a particular organization. Although the labels of licensing vs certifying boards are unfortunately homonyms, the vastly different powers become apparent with sociobioecological modeling.

The national certifying boards assess individuals for membership by ascertaining competency through training, testing, and on-going education/evaluation. Those individuals who successfully pass and maintain the process of certification—established by these certifying boards—are simply deemed as certified. Although the nomenclature of the ABPS may be interpreted as a taxonomical hierarchy more powerful than a state licensing board, this certifying organization possesses only the authority limited to determining who can possess its imprimatur. Board certified individuals—as well as those without certification granted by these certifying boards—are guided simply by the law of the land regarding the execution of skills. Individuals practice their craft based on licensure not certification. Certification is granted by organizations to individuals but the actual permission to legally practice a craft comes from the license granted by the state on the government society level. The certifying boards focus messaging on patient safety at the governmental society level. Whereas those who have earned certification often create societies (eg, ASPS or the American Society for Aesthetic Plastic Surgery) which require members to hold/maintain certification while imposing other criteria to join their fellowship. These societies perform lobbying activities not only extolling virtues of membership to the public but also directed at healthcare practitioners. Sandwiched in-between the individual and government society spheres of influence, however, is the community level (licensed facilities such as hospital, nursing home, surgical center) which determine their own rules for limiting scope of practice while following state law. Thus, on the individual level, healthcare practitioners freely perform procedures in private offices without limitation if they do not violate the licensing laws nor commit acts negligently. Actual restrictions are otherwise few and not aggressively policed. Restrictions are more frequently imposed as part of discipline after an adverse outcome. The certifying boards and societies in the organizational sphere are unable to directly restrict trade. On the other hand, through these same membership requirements, certifying boards and societies add an additional layer of policing for those practitioners who elect to join. In fact, some certifying boards require members belong to licensed facilities at the community level—or members may electively choose to practice in these facilities for logistical reasons of patient care—thereby subjecting practitioners to further regulatory scrutiny.^12^

The by-laws at facilities at the community level follow state regulations but show significant historical bias and favoritism toward the “established” practice of medicine through granting privileges based on credentialing processes. In theory based on state licensure, nothing should prevent a licensed nurse practitioner on the hospital staff from admitting patients or a general surgeon who feels comfortable from performing an implant-based breast reconstruction but for political, not legal, reasons. One of the largest accreditation organizations, The Joint Commission (JC), offers accreditation for numerous healthcare organizations including hospitals. This accreditation is a condition for reimbursement from payers. While JC does not specifically direct hospitals to mandate board certification of their medical staff, JC's overall standard for credentialing provides that privileging utilizes an objective, evidence-based process. Board certification is most often relied upon as the metric to provide objective criteria.^13^ Numerous class action lawsuits have been filed unsuccessfully against certifying boards for restriction of trade.^14–17^ Certification and licensure are vastly different.

**Figure 2.**
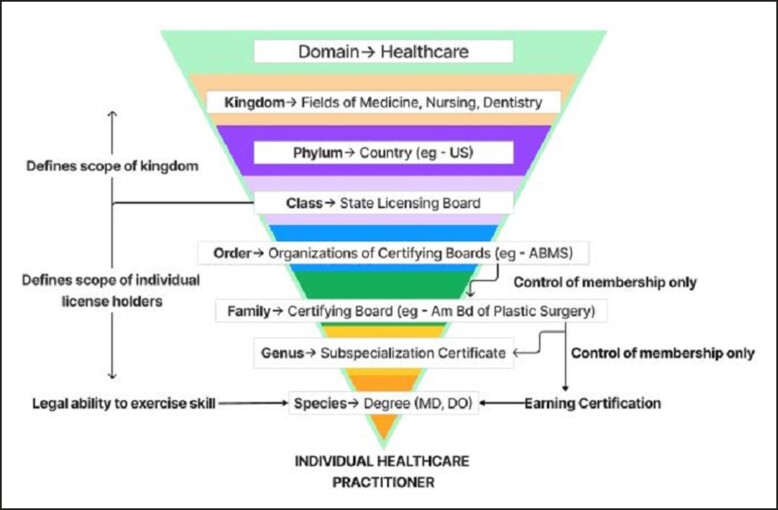
Taxonomic hierarchy of an individual healthcare practitioner exercising Plastic Surgery skillset in the United States.

To complicate matters, many of the certifying boards for physicians (eg, ABPS) have joined a larger entity known as the American Board of Medical Specialties (ABMS). There are 24 certifying boards that compose the ABMS. Member certifying boards of the ABMS, however, are not the only organizations that certify physicians. The American Board of Physician Specialties offers certificates to members who meet their specified criteria. In fact, the American Board of Physician Specialties grants certificates to both allopathic and osteopathic physicians. These certificates are also recognized by the Centers for Medicare and Medicaid Services, therefore demonstrating equivalency.^18^ Whereas some states only allow those certified by a board recognized within ABMS to call themselves board certified, other states do not have these restrictions. Similarly, hospital by-laws vary in their recognition of non-ABMS boards. Furthermore, the American Osteopathic Association administers its own array of recognized Specialty Certifying Boards in many fields (eg, Dermatology, Emergency Medicine, Surgery, etc) for those who trained in osteopathic programs.^19^Figure 2 maps the complex ecosystem using a hierarchical taxonomic methodology with various interactions between these entities for plastic surgeons.

## CONTROL OF A SPECIFIC BODY REGION OR TECHNIQUE: BATTLES FOR SCOPE OF PRACTICE

The natural evolution of healthcare is that those who practice in an isolated body part eventually fight for domination within that region as members pursue their scope of practice for economic survival through the conquest of renumeration. Although adaptations are made on an individual basis, selective pressures exist such that one organization dominates within overlapping scopes of practice at the family taxonomic level (ie, certifying board). In evolutionary biology, there is rarely enough substrate (prey) to support multiple different families, genera, or species seeking the same organism in the food chain over generations. This leads to evolutionary selection pressures such that species evolve a specific niche either through convergence or divergence so that they may each thrive separately. Failure to do so leads to extinction. Healthcare is no different.

### Divergent Pressures

One example of divergent evolution is how Otolaryngology entered the neck with thyroid surgery in the late 1950s; cosmetic facelifts in the 1970s; and ultimately neurosurgery (acoustic neuromas) in the 1980s.^20^ Although the family of certifying boards representing Otolaryngology (American Board of Otolaryngology Head and Neck Surgery, ABOto) and General Surgery (American Board of Surgery, ABS) both belong to the order ABMS; they are nevertheless completely different—hence competing as they function within the same scope. As Otolaryngology evolved dominance over general surgery in the neck, divergent evolution of these taxonomic families for mutual survival became apparent at the organizational level.

Other examples in organized medicine demonstrate these same evolutionary selection pressures. In Europe, breast reconstructive surgery is commonly performed by individuals trained in oncoplastic techniques, who otherwise do not possess plastic surgery monikers.^21,22^ Breast surgeons tend to fill this niche. Oncoplastic reconstruction is a growing divergent species.

### Convergent Pressures

Hand surgery provides a successful example of convergent evolution on an organizational level for a particular body part. Various fellowships after basic residency training exist for surgeons from such diverse families as orthopedic, plastic, or general surgery.^23^ Surgeons collegially coexist with this certificate of added qualification (CAQ) which is now known as a subspecialty certificate. This subspecialization is taxonomically at the genus level of the hierarchy. This model of additional training evolved through a cooperative process offered by certifying boards belonging to the same taxonomic order, ABMS. Unfortunately, for the species, on an individual level, this model adds additional training and testing beyond basic residency. This creates a significantly greater personal burden with the need for more training, testing, and expenses. However, from an organizational perspective, there is an advantage of quality and branding. Moreover, it advances patient care due to a diversity of expertise approaching a targeted and vulnerable community. Despite the individual negatives of the added burden, it is a positive evolutionary development for survival of the various certifying boards pursuing the same scope.

Wound care serves as another example of convergent evolution with respect to a similar scope but not necessarily limited to a specified body region. In sociobioecological modeling, convergent evolution facilitates cooperation and symbiosis.^24^ There are many healthcare practitioners from various taxonomic specialties, including members of differing kingdoms, who are qualified to perform wound care in some fashion. Multidisciplinary wound care centers, existent at most hospitals, demonstrate how various specialties cooperate for the benefit of the patient.

Clearly when the overlapping scopes are laborious, the fields performing these types of procedures narrow due to the lengthy training and copious energy required to exercise the skill. Recognizing the diverse backgrounds of those performing microsurgical anastomoses, membership in the American Society for Reconstructive Microsurgery is not limited to only plastic surgeons.^25^ As outlined, although a society differs from a certifying board, the broad membership reveals convergence. Moreover, it is not uncommon to see training seminars offered to healthcare providers performing microsurgical reconstruction from diverse backgrounds.^26^ Those who wish to seek skills in microsurgery regardless of order appear unhindered.

Examples abound outside of plastic surgery of convergent evolution at the genus level with subspecialty certification from the ABMS order. Sports medicine is a subspecialty certification offered by several participating ABMS certifying boards. Absent from this group though is orthopedic surgery. On the surgical side of this topic, however, the American Board of Orthopedic Surgery offers its members a subspecialty certification in Orthopedic Sports Medicine. Adolescent medicine, geriatric medicine, hospice and palliative medicine, pain medicine, and sleep medicine to name but a few are other subspecialty certifications, demonstrating successful convergent evolution for survival at the genus level through cooperation among the orders between different certifying boards of the ABMS. There is, however, a significant cost to practitioners at the individual level for the maintenance of these memberships.

## NUTRITIONALLY RICH RENUMERATION: AESTHETIC/COSMETIC SURGERY

Other techniques, such as aesthetic/cosmetic procedures, do not lend themselves to facile convergent evolution, as their scope is pursued by too many different species due to rich nutritional value (ie, renumeration). This potentially metamorphoses an ecosystem into the “Wild West.”

**Figure 3.**
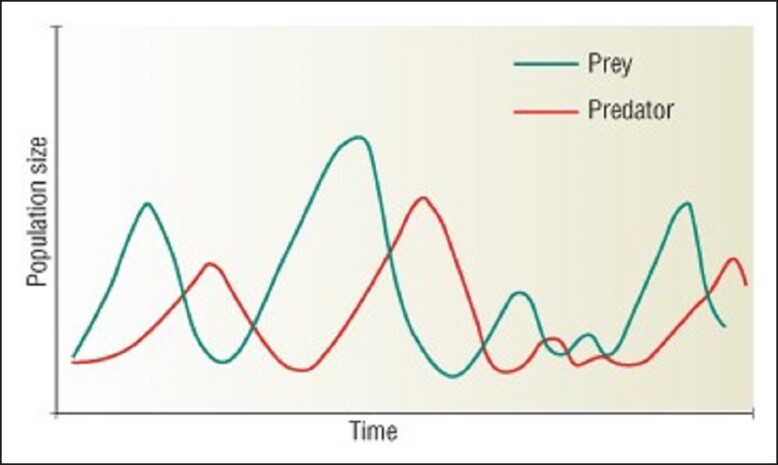
Classic Predator-Prey ecological interactions over time.

For example, there is growing, academic literature supporting the ability of Oral Maxillofacial Surgeons who belong to the American Board of Oral and Maxillofacial Surgery (ABOMS) to execute facelifts.^27,28^ The ABOMS is not in the same order as plastic surgeons nor otolaryngologists. Furthermore, the ABOMS offers its own subspecialization certificate in Head and Neck Oncologic and Reconstructive Surgery^29^ as well as Pediatric Craniomaxillofacial Surgery.^30^ These represent different genera entirely. Those who argue members of the ABOMS are “just dentists”—and hence in a different kingdom—ignore the fact that although pathways exist to earn certification from ABOMS with only a dental degree, the training nevertheless is through ABOMS recognized programs. Furthermore, given the current cultural climate of inclusivity, this exclusionary attitude—calling these practitioners “copycats”—rings of elitism and privilege. Nothing constructive is achieved by this strategy. Similar arguments regarding ability to perform aesthetic procedures with specified training are put forth by providers from other kingdoms that evolved separately: Advanced Practice Registered Nurses, Physician Associates, or Optometrists. Until recently in Nevada, dental hygienists administered injectables, provided they were under the supervision of a dentist.^31^ In Texas, although a strict set of rules are in place regarding medical supervision and record management, the actual injector needs only appropriate training to perform the procedure in a salon or spa.^32^ There is mission creep by different taxonomic domains entirely into aesthetic/cosmetic procedures. This interaction resembles the Lotka–Volterra Model of Predator–Prey relationships which is mapped in Figure 3.^33^

In the background, there is evidence of potential cannibalization even within the ABMS, as the ABPS and ABOto cooperatively achieved approval—but not yet issued—further subspecialty certifications for plastic surgery within the head and neck.^34^ This additional silo of subcertification at the genus level of taxonomy has the potential to flank its own membership undoubtedly complicating an already crowded field. Also within the ABMS, some members of the American Board of Dermatology not only receive training in the use of Botox and/or fillers within the face^35^ but also in tumescent liposuction. Similarly, members of the American Board of Family Medicine or Internal Medicine receive training in certain surgical procedures.^36^

As these turf battles rage for aesthetic/cosmetic procedures, the American Board of Cosmetic Surgery,^37^ the American Board of Facial Plastic and Reconstructive Surgery,^38^ and the American Board of Facial Cosmetic Surgery,^39^ all offer certifying examinations following their own recognized training pathways. Those earning any of these additional certifications can honestly message their double-boarded status, hence eliminating a competitive advantage once held by plastic surgeons who completed the Independent Pathway to certification.^40^ On the community level, however, whether this second non-ABMS certification is recognized becomes irrelevant as practitioners possess a primary ABMS certification.

An ongoing discussion in vascular surgery illustrates the grave consequences of ignoring evolution on an organizational level and its downstream ramifications at the community level. The Vascular Surgery Board remains part of the ABS. This complicates the development of training programs in vascular surgery or tailoring the certification process for those who exclusively and independently practice vascular surgery. This restriction has been severely problematic, as the advancement of interventional procedures increases the capabilities of competing practitioners. Consequently, those trained in Cardiology (American Board of Internal Medicine) and Interventional Radiology (American Board of Radiology) significantly overlap in scope with vascular surgeons.^41^ Vascular surgeons are potentially placed at a competitive disadvantage within their own orders. This results in a lost opportunity. Attempts by the Vascular Surgery Board to separate from the ABS—becoming independently recognized—continues to fail.^42–44^ As a result, vascular surgeons are losing the battle to exclusively perform endovascular procedures at the community level.^45^ This serves as a stark warning that although healthcare practitioners from different families possess varying skills, when performing the same procedure, evolutionary principles are forefront; not who believes he or she can execute the procedure better.

For vicious turf battles to perpetuate between competing organizations, 2 components must be present: (1) monetary renumeration significant enough to finance interest (nutritional value of the target) and (2) belief no further CAQ-type training is required but for graduation from a program that is a member of a board claiming expertise while operating legally without negligence (skillset of the pursuer). Therefore, the logical conclusion from an evolutionary standpoint is that the turf battle for aesthetic/cosmetic procedures involving injectables performed in the office will be lost by the plastic surgeons due to the sheer volume of professionals from vastly different taxonomic hierarchies claiming expertise in the same scope. To think messaging alone will direct patient flow in this arena is naïve. Additionally, from a strategic standpoint, throwing limited resources at the injectable scope is wasteful. There are simply too many competing healthcare professionals. If one of the required components to instigate a turf battle is eliminated—satisfactory renumeration—a conflict is much less likely to occur.^46,47^ Renumeration from injectables simply does not warrant a turf battle on an organizational level.

## SOLUTIONS

As plastic surgery is ultimately a reproducible skill potentially achievable by any practitioner who chooses to study it, how can it be uniquely branded on an organizational level?^48^

### Individual-Level Solutions

Some confidently argue on an individual level that hard work will produce a successful practice and lead to survival.^49^ This works if an individual outcompetes other healthcare providers for resources at the community level. But if the goal is the survival of an organization as a taxonomic family, this is a poor strategy. Individual-level actions do not address overall long-term survival of an organization. Leveraging modern marketing techniques, such as social media influencers, is unlikely to alter the long-term survival of plastic surgery on an organizational level. Nothing prohibits healthcare providers, regardless of training, from promoting a procedure that they are legally permitted to perform. Similar hard-working adaptations could be exercised by others on an individual level in competing fields of different orders and kingdoms, thereby perpetuating the evolutionary struggle. Therefore, to end this battle in a single lifetime instead of generations, rapid organizational evolution is required.

### Radical Step: Restorative Healthcare

A radical step is needed to solve this branding problem for organizational survival in plastic surgery. Turning toward the sociobioecological perspective, perhaps messaging failed because the Darwinian assumption that DNA/genes (or professional training) provide an evolutionary advantage is wrong. Perhaps the public only sees homogeneity in the taxonomic hierarchy of various healthcare specialties, including kingdoms. Perhaps on the granular level, it is clinician behavior based on culture, that is, the true determinant of organizational survival while maintaining legal boundaries.^50^ Therefore, a culture change among plastic surgeons is required. Establishing a radically new specialty at the domain level solves this.

Restorative healthcare achieves the goal. The term restorative implies returning, repairing, and renovating. This label incorporates both reconstructive and cosmetic endeavors. Moreover, it includes the budding fields of Preventive and Regenerative Medicine, a growing field of science. Other areas addressed by this revolutionary change include reconstructive transplantation, tissue engineering, wound healing, and nerve regeneration.^51,52^ As stewards of this field, membership in restorative healthcare is not limited to only restorative surgery or restorative medicine. Diverse leadership from different hierarchical kingdoms would work cooperatively in parallel to design and grant certification within their own class. Certification of those members from Nursing, Dentistry, and Optometry would be achieved by granting subspecialty certificates at the genus level while meticulously maintaining legal recognition of licensure limits. The focus would be on the mission of scientific advancement to improve patient outcome and safety, not protection of turf. There is opportunity for the growth of a technology arm of this new kingdom, whereby opportunities in Artificial Intelligence and Nanotechnology are explored. This dramatic move anticipates the evolutionary direction of an entire field. It accounts for the diversity in training and skills of those seeking similar scopes yet respects barriers established legally at the government society level. Branding would start at the nascency of this new field's birth, rather than trying to catch up after years of evolution in a crowded ecosystem. As recognized leaders, symbiotic participants will naturally grow the field. This would operationalize the new kingdom toward having another member earn a Nobel Prize, as did Dr J Murray.^53^

Arguing that this change is simply unrealistic or too complicated disregards the likely possibility of organizational extinction for plastic surgery within the next few generations. Those who think financing is unavailable ignore the drive by private equity toward return on investment.^54,55^ As the Flexner Report revolutionized medical education with an extinction-level event for many medical schools,^56^ a similar meteoric impact is required to advance plastic surgery.
